# Stability investigations of cytochrome P450 (CYP) enzymes immediately after death in a pig model support the applicability of postmortem hepatic CYP quantification

**DOI:** 10.1002/prp2.860

**Published:** 2021-09-03

**Authors:** Kata W. Pedersen, Jakob Hansen, Jørgen B. Hasselstrøm, Jakob R. Jornil

**Affiliations:** ^1^ Department of Forensic Medicine Aarhus University Aarhus Denmark

**Keywords:** CYP1A2, CYP2D25, CYP2E1, CYP3A29, cytochrome P450, drug metabolism, enzyme, PBPK, postmortem

## Abstract

Quantification of drug‐metabolizing cytochrome P450 (CYP) isoforms using LC–MS/MS has been proposed as a potential way of estimating antemortem CYP levels using postmortem tissue, but the postmortem stability of CYP proteins is incompletely investigated. If one can use data obtained from the analysis of postmortem specimens to inform physiologically based pharmacokinetic (PBPK) models this greatly increases the access to rare specimens among special subpopulations. In this study, we developed and validated an LC–MS/MS method for targeted CYP protein quantification in a porcine animal model to study postmortem stability. We measured 19.9–28.3 pmol CYP1A2, 50.3–66.2 pmol CYP2D25, 132.9–142.7 pmol CYP2E1, and 16.8–48 pmol CYP3A29 protein per mg PLM in nondegraded tissue. In tissue stored at 4°C, we found that the CYP protein levels were unaffected by degradation after 72 h. At 21°C CYP1A2, CYP2D25, and CYP2E1 protein levels were nearly unaffected by degradation after 24 h, whereas a loss of approximately 50% was seen after 48 h. At 21°C CYP3A29 had a loss of 50% at 24 h and 70% at 48 h exhibiting less postmortem stability. In vitro enzyme activity measurements in the same tissue stored at 21°C showed a 50% decrease after 24 h and a complete loss of enzyme activity after 48 h. When stored at 4°C, the in vitro enzyme activity decreased to 50% activity after 96 h. In conclusion, measuring CYP levels by an LC–MS/MS approach was clearly less affected by postmortem changes than an activity‐based approach. The found postmortem stability for 24 h at 21°C for 3 out of 4 CYP isoforms supports the use of properly stored postmortem tissue to inform PBPK models.

## Graphical abstract

This study investigates the postmortem stability of porcine CYP1A2, CYP2E1, CYP2D25, and CYP3A29 using LC‐MS/MS. Our findings suggest that postmortem hepatic tissue can be used to estimate antemortem CYP levels.

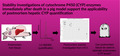

**Abbreviations**
ACN: Acetonitrile
AmBic: ammonium bicarbonate
AQUA: absolute quantification
BSA: bovine serum albumin
CE: collision energy
CV: cone voltage
CYP: cytochrome P450
FA: formic acid
HLMs: human liver microsomes
IS peptides: internal standard peptides
LC–MS/MS: liquid chromatography–tandem mass spectrometry
MMTS: methyl methanethiosulfonate
MPPGL: microsomal protein per gram liver;
PLM: porcine liver microsomes
PMI: postmortem interval
SDC: sodium deoxycholate
SIL‐IS peptides: stable isotope‐labeled internal standard peptides
TCEP: tris(2‐carboxyethyl)phosphine

## 1 INTRODUCTION

Physiologically based pharmacokinetic (PBPK) models are increasingly being used to predict human drug disposition across subpopulations.^1^, ^2^, ^3^ Hepatic drug metabolism by the cytochrome P450 (CYP) family of enzymes is a major elimination pathway for a large number of drugs and thus affects drug disposition. Therefore, information regarding CYP levels in subpopulations is needed. There are numerous examples of subpopulations with altered CYP levels, to mention a few: Obese have reduced CYP3A activity with increasing bodyweight^4^; Pregnant have increased activity of CYP2D6 and CYP3A4 and reduced CYP1A2 activity^5^; Individuals suffering from cirrhosis have markedly reduced activity of CYP1A2 and CYP3A4.^6^

A limitation in the aforementioned studies is that in vitro or in vivo CYP‐activities have been used to estimate altered CYP protein levels in a given subpopulation. The apparent change in CYP activity in a subpopulation might be a result of different CYP protein levels in combination with e.g., altered drug transporter levels and altered blood flow. Therefore the true altered CYP protein levels in a given subpopulation might not be known. Access to relevant hepatic tissue is scarce. If CYP protein levels could be estimated in postmortem hepatic tissue taken at an autopsy, this would dramatically ease the access to relevant hepatic tissue for special subpopulations, but also raise the question: What is the postmortem timeframe in which CYP protein levels reflect antemortem levels?

Tissue for proteomic research is generally snap‐frozen in, for example, liquid nitrogen (−196°C) as quickly as possible to avoid protein modification by oxidation, proteolysis, and bacterial degradation. On the contrary individuals in a postmortem setting might have been dead for several days before autopsy and the quality of this tissue for protein quantification can be questioned. Investigations on postmortem tissue found that some proteins are susceptible to modification or degradation during the first 24 h postmortem, whereas other proteins are stable for days after death.^7^, ^8^, ^9^, ^10^ LC–MS/MS‐based quantification of CYP protein levels in postmortem hepatic tissue with values similar to reported reference levels has been reported,^11^ but the investigated hepatic tissue had postmortem intervals (PMIs, time from death to autopsy) between 24 and 36 h. Therefore there is currently a knowledge gap regarding hepatic CYP stability in the first 24 h postmortem.

We chose a pig model to study the postmortem stability of CYP proteins. Pigs are highly suitable to study human biotransformation due to the similarities in anatomy and physiology and a good model for studying postmortem protein degradation.^9^, ^12^ Many porcine CYP enzymes exhibit more than 80% sequence identity to human drug‐metabolizing CYP enzymes,^13^, ^14^ and the enzyme activities of key porcine CYP subfamilies have been studied in vitro with probe substrates commonly used for human studies.^15^, ^16^ Furthermore, porcine CYP enzymes are evenly expressed in the lobes of the liver and at similar levels as human CYP enzymes.^17^, ^18^, ^19^

As a first step to justify using CYP quantification data from forensic postmortem hepatic tissue in PBPK modeling, the aim of the current study was to investigate the postmortem stability of CYP enzymes immediately after death and up to 7 days in a porcine model. We developed a quantitative LC–MS/MS proteomics method based on the AQUA quantification strategy^20^ targeting four porcine CYP isoforms, CYP1A2, CYP2D25, CYP2E1, and CYP3A29, which exhibit high sequence identity (>76%) to human CYP1A2, CYP2D6, CYP2E1, and CYP3A4/5, respectively (Table S1). We used the developed method to quantify the CYP proteins in porcine liver microsomes (PLM) from nondegraded hepatic tissue (PMI = 0) and hepatic tissue degraded in vitro at 4°C and 21°C (PMI = 2–168 h). Finally, we wanted to gain further insight into the stability of CYP enzymes by investigating the in vitro enzymatic activity based on the metabolite formation associated with phenacetin and midazolam metabolism. To our knowledge, this is the first study investigating the postmortem stability of CYP enzymes in hepatic tissue 0–24 h and the first quantification of porcine CYP1A2, CYP2D25, CYP2E1, and CYP3A29 using targeted mass spectrometry.

## 2 METHODS AND MATERIALS

### 2.1 Reagents and solutions

Reagents were purchased from Sigma‐Aldrichunless otherwise stated. Methyl methanethiosulfonate (MMTS) was dissolved in 2‐propanol at a concentration of 80 mM, and TCEP tris(2‐carboxyethyl)phosphine (TCEP) was prepared as 35 mM in 1.5 M ammonium bicarbonate (Ambic). Paracetamol was purchased from Merck. Midazolam was from Toronto Research Chemicals, and alpha‐hydroxymidazolam was from Cerilliant. The NADPH regenerating system (RAPID start K5100) was from XenoTech. The Bradford protein assay for MPPGL determination was from Bio‐Rad, and TPCK‐treated trypsin for protein digestion was from AB‐SCIEX. Ultrapure water (type 1) was obtained from a Milli‐Q IQ 7000 apparatus (Millipore).

### 2.2 Porcine liver samples and stability assay

Porcine livers from Danish slaughter pigs (*Sus scrofa domesticus*) (*n* = 3, female, 6 months old) were placed on ice immediately after sacrifice. The pigs were cared for according to the Danish Animal Welfare Act 2013. Furthermore, the animals were sacrificed as part of meat production before entering our study, and therefore no ethical approval was needed according to the Danish ministerial order on animal experimentation (BEK nr.2028 of 14/12/2020). For each liver, approximately 2 g of tissue was frozen at −80°C, and these samples were annotated as *t* = 0. Large pieces (>1 kg) from each liver were stored in plastic bags and incubated at 4 or 21°C before the samples (~2 g) were dissected and frozen at *t* = 2, 4, 6, 20, 24, 48, 72, 96, 120, 144, or 168 h. The liver pieces at 4°C were sampled for up to 7 days (168 h), whereas the liver pieces at 21°C were only sampled up to 72 h due to advanced putrefaction.

### 2.3 Preparation of porcine liver microsomes

Liver tissue (500 mg) was thawed and minced with a scalpel. The minced tissue was added to 3 ml of cold homogenization buffer (0.1 M potassium phosphate buffer with 0.125 M potassium chloride and 1 mM EDTA, pH 7.5) and homogenized using a Potter‐Elvehjem glass homogenizer (VWR) and a Teflon pestle which was driven by a RW16 motor unit (IKA) at speed level 6. The homogenate was centrifuged at 9000*g* for 20 min at 4°C to generate the S9 fraction. The S9 fraction supernatant was transferred to 10.4 ml polycarbonate centrifugation tubes (Beckman Coulter) and centrifuged at 100,000*g* for 75 min at 4°C using an Optima L‐90K ultracentrifuge (Beckman Coulter, Inc.). The microsomal pellet was dissolved in 200 µl of microsomal storage buffer (50 mM phosphate buffer with 250 mM sucrose, pH 7.5) and stored at −80°C until further use. The microsomal protein concentration was determined by Bradford protein assay using a bovine serum albumin (BSA) standard curve. An in‐house postmortem PLM pool together with a commercial HLM pool (Ultrapool HLM 150, BD Biosciences) were used for quality control. Microsomal protein per gram liver (MPPGL) was calculated as the determined microsomal protein concentration divided by the amount of liver used. MPPGL is not corrected for process loss.^21^ The MPPGL measurements were used to adjust the PLM concentrations, ensuring that equal amounts of microsomal protein from each sample preparation were used for CYP quantification and enzyme activity measurements.

### 2.4 Peptide selection

Tryptic peptides suitable for LC–MS/MS quantification based on the AQUA strategy^20^ were selected based on information from PeptideAtlas (ISB),^22^ Skyline 19.1 peptide prediction software,^23^ and the literature.^24^ For each porcine CYP protein, four to six unique synthetic peptides (SpikeTides, JPT) were manually tuned by direct infusion into the mass spectrometer and subsequently evaluated in terms of signal intensity and linearity. The two best‐performing peptides for each CYP protein were acquired as quantified synthetic analog stable isotope‐labeled internal standard peptides (SIL‐IS peptides) containing C‐terminally isotopic labeled Arg (^13^C_6_^15^N_4_) or Lys (^13^C_6_^15^N_2_) (SpikeTides TQL, JPT) (Tables S1 and S2).

### 2.5 Trypsin digestion

The isolated PLM were subjected to enzyme digestion following a previously described protocol, with minor deviations.^11^ In brief, all PLM preparations were diluted in microsomal storage buffer to a working solution of 7 µg PLM/µl. In Eppendorf LoBind tubes, 10 µl of diluted PLM was added to 10 µl of H_2_O and 5 µl of IS‐peptides (200 fmol/µl 1A2, 200 fmol/µl 2D25, 600 fmol/µl 2E1, and 200 fmol/µl 3A29 dissolved in 0.1 M AmBic with 20% acetonitrile (ACN) added according to the manufacturer's instructions). For denaturation, 5 µl of a 12% sodium deoxycholate (SDC) solution was added to the mix and incubated at 80°C for 10 min with vigorous rotational shaking. The denatured mix was cooled to 60°C, and 5 µl of 35 mM TCEP was added, followed by incubation for 20 min at 60°C. The reduced proteins were cooled to room temperature and alkylated by incubation with 5 µl of 80 mM MMTS for 20 min. The solution was diluted with 70 µl of 25 mM AmBic, and 10 µl of trypsin mix (0.033 µg/µl trypsin prepared in 25 mM AmBic with 2 mM CaCl_2_, 1:30 w/w trypsin:protein) was added. The digestion proceeded for 4 h at 37°C, after which the reaction was stopped, and SDC was precipitated by the addition of formic acid (FA) to a final concentration of 0.3% FA. The solution was vortexed vigorously followed by centrifugation at 15,000*g* for 2 min, after which the supernatant was moved to a glass vial and analyzed by LC–MS/MS.

### 2.6 **LC**–**MS/MS analysis of peptides**

The digested peptides were analyzed using a triple quadrupole mass spectrometer (Waters Xevo TQ‐S) coupled with a UPLC (Waters Acquity). The targeted LC–MS/MS method was previously described and consisted of monitoring unique signature peptides along with SIL‐IS peptides containing either stable or isotopic labeled Arg (*R = R + 10 Da) or Lys (*K = K + 8 Da).^11^ The signature peptides are as follows: CYP1A2: YLPSPTLQR/YLPSPTLQR* (537.8 > 798.4)/(542.8 > 808.5); CYP2D25: DLAQPPR/DLAQPPR* (398.7 > 369.2)/(403.7 > 379.2); CYP2E1: FIDLIPSNLPHEATR/FIDLIPSNLPHEATR* (862.0 > 489.3)/(867.0 > 489.3); and CYP3A29: SSVNFFTK/SSVNFFTK* (465.2 > 755.4)/(469.2 > 763.4). Additional information including qualifier peptide sequences, m/z of the MRM transitions, cone voltage (CV), and optimized CE (collision energy) for the peptides used in this study are displayed in Tables S1 and S2 in the supplementary material. CYP concentrations were determined based on peak area response (PAR response) by AQUA.^20^

$$\text{PAR}\;\text{response}=\frac{\text{Area}\;\text{signature}\;\text{peptide}}{\text{Area}\;\text{SIL}‐\text{IS}\;\text{peptide}}$$

$${\text{pmol}}_{\text{signature}\;\text{peptide}}=\text{PAR}\;\text{response}\times {\text{pmol}}_{\text{SIL}‐\text{IS}\;\text{peptide}}$$

$$\text{[CYP]}=\frac{{\text{pmol}}_{\text{signature}\;\text{peptide}}}{\text{mg}\;\text{PLM}\;\text{protein}}$$

### 2.7 Method validation

#### 2.7.1 Precision

The precision of the method was evaluated using pooled PLM (pPLM) comprised of equal amounts of PLM from three porcine livers (*t* = 0). The pPLM were analyzed in triplicate on seven different days. The precision of the method was determined at low (25 µg) and high (70 µg) concentrations of pPLM. BSA was added to the low pPLM to adjust the protein level to the same level as that of the high pPLM. The precision was determined as within‐ and between‐runs variation calculated using a one‐way ANOVA approach.

#### 2.7.2 Trueness

The trueness of the method was evaluated at the peptide level by comparing the closeness of agreement between triplicate unspiked samples and triplicate samples spiked with a known amount of unlabeled synthetic target peptides. Trueness was measured at two spike levels depending on the endogenous level of the target peptide in the matrix: 1A2 (20, 10 fmol), 2D25 (20, 10 fmol), 2E1 (50, 25 fmol), and 3A29 (30, 15 fmol).

$$\text{Trueness}\;\left(\%\right)=\frac{\left[\text{spiked}\;\text{samples}]‐[\text{unspiked}\;\text{samples}\right]}{\text{spike}\;\text{concentration}}\times 100$$

#### 2.7.3 LOD/LLOQ

The limit of detection and lower limit of quantification of the method were determined based on the SIL‐peptide because no blank matrix exists. pPLM spiked with low concentrations of SIL‐peptides 1A2 (17 fmol), 2D25 (20 fmol), 2E1 (75 fmol), and 3A29 (40 fmol) were subjected to enzyme digestion, performed as three reactions per day on three different days (a total of nine reactions, CV <20%). The LOD was estimated as three times the SD of the reactions, and the LLOQ was estimated as ten times the standard deviation. The peak areas were converted into fmol by one‐point calibration using the average SIL‐peptide peak area from five separate samples.

### 2.8 Activity assay

The in vitro enzyme activity rates of porcine CYP enzymes in PLM were estimated by LC–MS/MS quantification of metabolite formation. The in vitro enzyme activity of CYP1A was estimated based on the metabolism of phenacetin (substrate) to its metabolite paracetamol, and the in vitro enzyme activity of CYP3A was estimated based on the metabolism of midazolam (substrate) to its metabolite alpha‐hydroxymidazolam. An NADPH regenerating system (RAPID start K5100, XenoTech) was diluted according to the manufacturer's instructions (final concentration in the reaction mixture: 1 mM NADP, 5 mM glucose 6‐phosphate, 1 Unit/ml glucose 6‐phosphate dehydrogenase, 3.3 mM MgCl_2_, and 0.1 M phosphate buffer) and preincubated for 5 min at 37°C with phenacetin and midazolam at final concentrations of 100 and 10 µM, respectively. To start the assay, PLM (diluted with 0.1 M phosphate buffer) was added to the mix to a final concentration of 1 mg PLM/ml. The reaction time was 20 min, after which the reaction was stopped by transferring 30 µl of the mixture to 70 µl of ice‐cooled ACN containing the internal standards midazolam‐D6 and 3‐acetamidophenol at final concentrations of 1.4 and 4.6 µM, respectively. The stopped reaction mixture was diluted 15‐fold in 10% ACN, and the metabolites were quantified by targeted LC–MS/MS analysis. The assay was validated and optimized in terms of reaction time (6–30 min) and PLM (enzyme) concentration (0.1–1.5 mg PLM). The two enzyme reactions were performed in one assay, as no interference was observed between the two reactions.

### 2.9 **LC**–**MS/MS analysis of metabolites**

The concentrations of paracetamol and alpha‐hydroxymidazolam were measured using a triple quadrupole mass spectrometer (Waters Xevo TQ‐S) coupled with a UPLC system (Waters Acquity). The samples were injected (7.5 µl) and separated on a C18 column (Waters UPLC HSS C18, 1.8 µm, 2.1 mm × 100 mm, column temperature = 40°C) with a solvent flow rate of 0.4 ml/min. The initial mobile phase conditions were 90% solvent A (H_2_O with 0.1% FA) and 10% solvent B (ACN with 0.1% FA). A gradient from 10 to 100% solvent B was performed over 3 min, after which 100% solvent B was maintained for one additional minute. In the following 4 min, the column was equilibrated to the initial mobile phase conditions. Electrospray ionization in positive mode was used, and the analytes were monitored with the following optimized MRM transition parameters: MRM1 = quantifier, MRM2 = qualifier: paracetamol (MRM1 = 152.0 > 110.2, CV/CE = 24/16 and MRM2 = 152.0 > 93.1, CV/CE = 24/23, retention time 1.15 min) and its internal standard 3‐acetamidophenol (MRM1 = 152.0 > 110.2, CV/CE = 24/16, retention time 1.33 min); alpha‐hydroxymidazolam (MRM1 = 342.1 >324.2 CV/CE = 24/19 and MRM2 = 342.1 >203.1 CV/CE = 24/26) and its internal standard midazolam‐D6 (MRM1 = 332.0 >297.0 CV/CE = 24/24 and MRM2 = 332.0 > 211.2 CV/CE = 24/31). The mass spectrometer was operated in multiple reaction monitoring (MRM) mode with a capillary voltage of 2.4 kV, source temperature at 150°C, desolvation temperature at 600°C and a desolvation gas flow (nitrogen) of 800 L/h. Metabolite formation was calculated using internal standards and calibration curves created with linear regression using a 1/*x* weighing factor, excluding zero. Calibration curves were based on 15 levels of paracetamol (0.0012–20 µM) and alpha‐hydroxymidazolam (0.0009–7.5 µM) prepared by diluting stocks of 21 µM paracetamol or 16 µM alpha‐hydroxymidazolam in 0.1 M phosphate buffer to the highest calibrator concentration, followed by twofold serial dilutions. The calibration curve was analyzed in the same matrix as the assay samples (pool of PLM), not including the NADPH generating system.

### 2.10 Calculation of confidence intervals

The 95% confidence interval (CI) of the mean value, of two or three different livers, at each time point was calculated using an assumption of equal variance at different time points. The standard deviation for the 95% CI was calculated as the within groups standard deviation using one‐way ANOVA.

### 2.11 Nomenclature of targets and ligands

Key protein targets and ligands in this article are hyperlinked to corresponding entries in http://www.guidetopharmacology.org, the common portal for data from the IUPHAR/BPS Guide to PHARMACOLOGY,^25^ and are permanently archived in the Concise Guide to PHARMACOLOGY 2019/20.^26^

## 3 RESULTS

### 3.1 Postmortem stability of microsomal proteins

**FIGURE 1:**
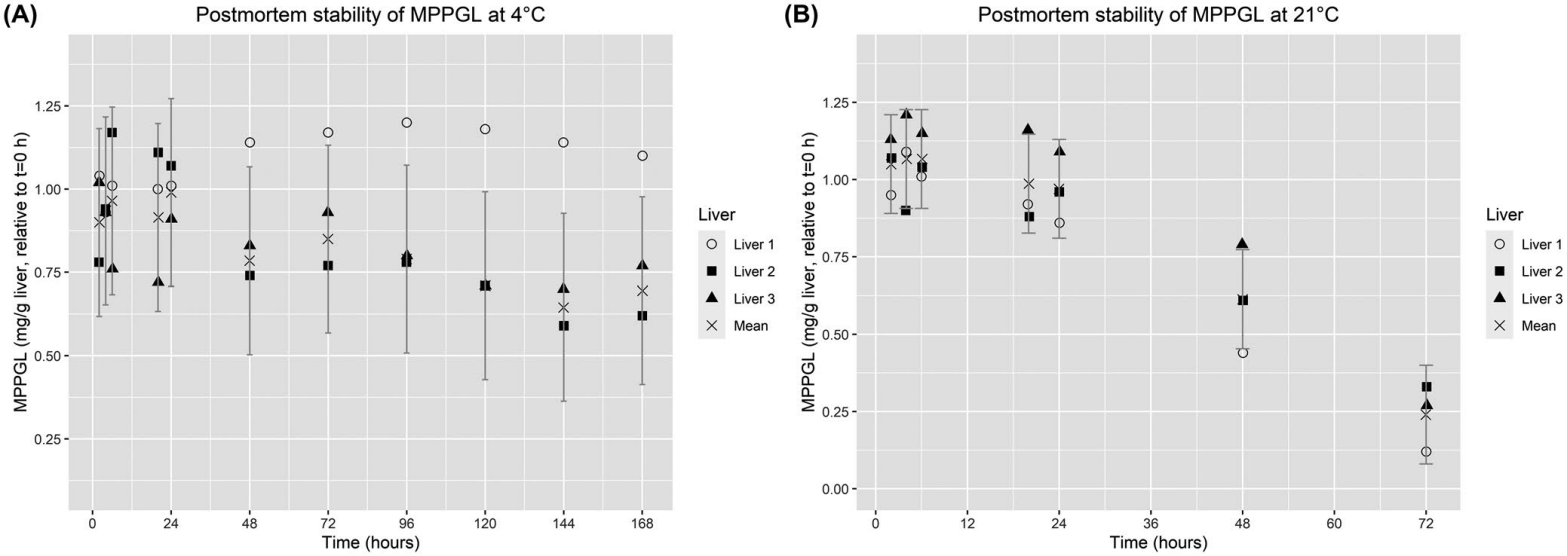
MPPGL yield during postmortem degradation at 4°C (A) and 21°C (B) in three porcine livers. Individual and mean microsomal protein content per gram liver (MPPGL) levels shown relative to *t* = 0 h. 95% confidence intervals shown as calculated by ANOVA for all three livers at 21°C and due to different degradation profiles only for livers 2 and 3 at 4°C

The yield of total microsomal protein (MPPGL) in liver 1 was stable for 168 h at 4°C (Figure 1). For liver 2 and 3 at 4°C the MPPGL levels were stable for approximately 96 h, after that a decline of approx. 30% was seen. When the tissues were stored at 21°C, the MPPGL levels were stable for at least 24 h. After 48 h of storage at 21°C, over 60% of the *t* = 0 levels were still measurable, whereas at 72 h, the MPPGL levels were severely affected by putrefaction and less than 25% of the *t* = 0 levels were detected. The 95% CI in Figure 1 was at 4°C calculated using liver 2 and 3 since liver 1 had a different degradation profile, at 21°C all three livers were used to calculate the 95% CI. The absolute levels of MPPGL are shown in Table 1. To assess the variation in the PLM preparations, triplicate PLM preparations were performed on two livers at *t* = 0. The purifications yielded an average of 14.1 ± 1.5 (CV: 10.8%) and 10.9 ± 1.5 (CV: 13.4%) MPPGL for the two livers.

**TABLE 1:** CYP protein expression levels and activity rates in nondegraded porcine liver tissue. The CYP protein levels and the enzyme activity rates are reported as the mean of two PLM (*t* = 0) preparations from each liver

	MPPGL^a^ (mg/g)	Protein level (pmol/mg PLM)				Metabolite formation rate (pmol/min/mg PLM)	
		CYP1A2	CYP2D25	CYP2E1	CYP3A29	Phenacetin	Midazolam
Liver 1	10.9	21.6	68.5	143	16.8	3800	2300
Liver 2	14.6	28.3	50.3	139	48.5	6000	4000
Liver 3	11.4	19.9	66.2	133	34.1	1600	1000

^a^ Microsomal protein per gram of liver at *t* = 0 h.

### 3.2 Validation of LC–MS/MS method

To investigate the postmortem stability of CYP proteins specifically, we developed a LC–MS/MS method for quantification of four CYP protein isoforms in PLM based on quantification of unique signature peptides (Table S1). The developed method was validated in a nondegraded matrix (*t* = 0) in terms of method precision, method recovery, trueness of the method, LOD, and LLOQ. The performances of the quantifying peptides are presented in Table 2.

**TABLE 2:** Validation of the LC–MS/MS method for CYP quantification

Target peptide	1A2_1	2D25_1	2E1_1	3A29_1
	YLPSPTLQR	DLAQPPR	FIDLIPSNLPHEATR	SSVNFFTK
Precision				
High sample level				
Within—run (CV %)	7.1	2.9	3.4	5.4
Total (CV %)	12.2	14.1	6.2	8.8
Low sample level				
Within—run (CV %)	12.8	8.4	7.9	3.7
Total (CV %)	14.1	12.3	8.4	13.8
Trueness % (high spike level)	102.8	106.1	119.8	114.1
Trueness % (low spike level)	94.6	100.3	114.1	98.9
LOD (fmol CYP/µg PLM)	0.06	0.15	0.03	0.82
LLOQ (fmol CYP/µg PLM)	0.19	0.49	0.11	2.74

### 3.3 Stability of CYP proteins in postmortem hepatic tissue

**FIGURE 2:**
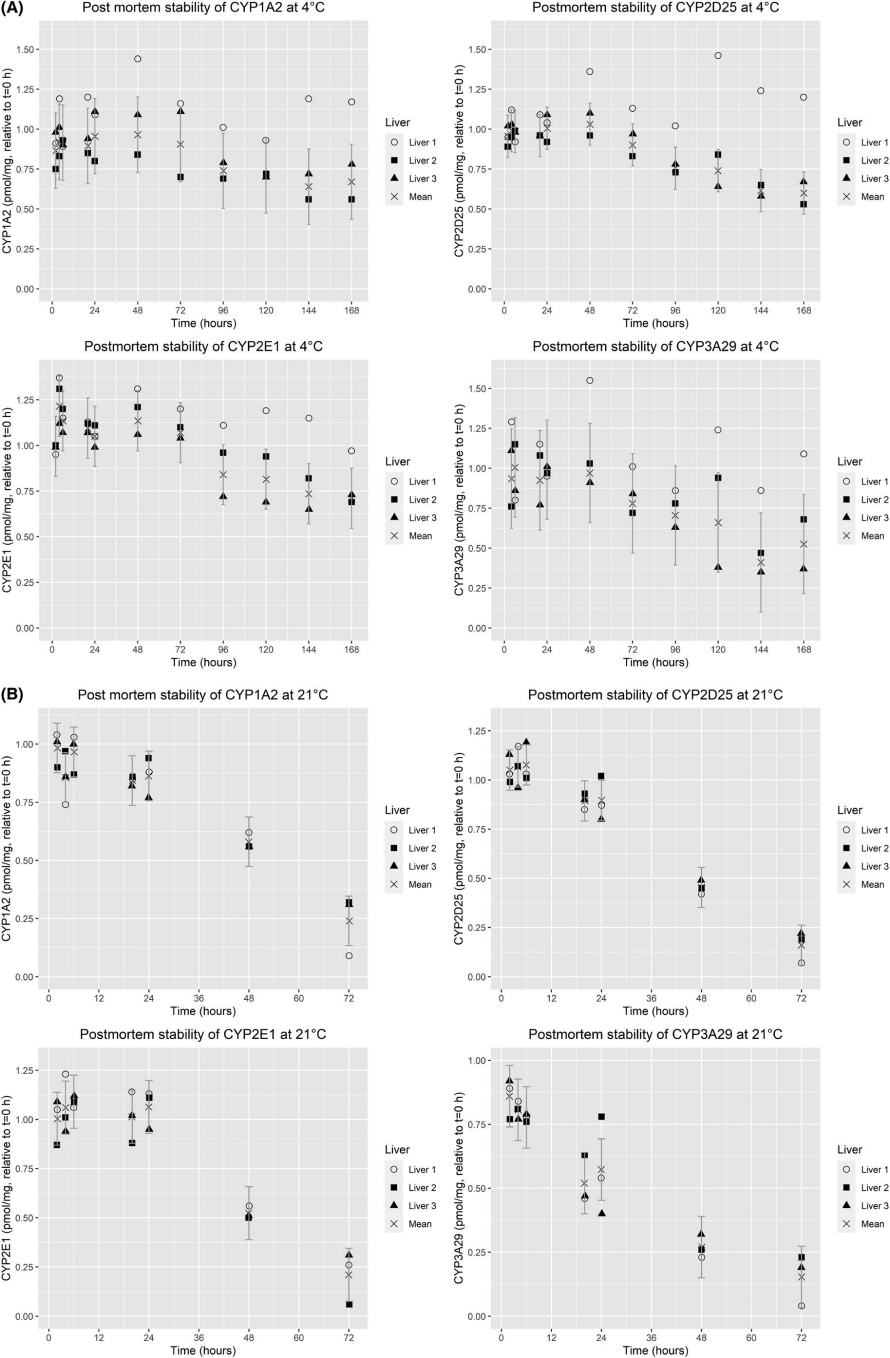
(A) CYP1A2, CYP2D25, CYP2E1, and CYP3A29 PLM concentrations in postmortem hepatic tissue at 4°C. Individual and mean cytochrome P450 (CYP) concentrations in porcine liver microsomes (PLM) relative to *t* = 0 h. 95% confidence intervals shown as calculated by ANOVA for livers 2 and 3, since liver 1 had a different degradation profile. Each PLM preparation was analyzed as technical duplicates by LC–MS/MS. (B) CYP1A2, CYP2D25, CYP2E1, and CYP3A29 PLM concentrations in postmortem hepatic tissue at 21°C. Individual and mean cytochrome P450 (CYP) concentrations in porcine liver microsomes (PLM) relative to *t* = 0 h. 95% confidence intervals shown as calculated by ANOVA for all three livers. Each PLM preparation was analyzed as technical duplicates by LC–MS/MS

At 4°C a similar pattern to MPPGL stability was seen for CYP1A2, CYP2D25, CYP2E1, and CYP3A29 (Figure 2A) with a high degree of stability from 0 to ~72 h at 4°C for liver 1–3, and a decrease of 30–50% in CYP levels for liver 2 and 3 beyond 72 h. Liver 1 had stable CYP levels for all 168 h. The protein levels of CYP1A2, CYP2E1, and CYP2D25 were reasonably stable at 21°C for 24 h (Figure 2B). When the tissue was stored at 21°C for 48 h, a significant loss in CYP protein content was observed and approximately 50–60% of the *t* = 0 h levels were still measurable after 48 h for these three CYP isoforms. For CYP3A29 at 21°C a different profile was seen. During the first 6 h there was no major decrease, but after 20 h a decline of approximately 40%–50% was seen. The 95% CI at 4°C (Figure 2A) was calculated using liver 2 and 3 since liver 1 had a different degradation profile, at 21°C (Figure 2B) all three livers were used to calculate the 95% CI. The absolute concentrations of the CYP isoforms are shown in Table 1.

### 3.4 Enzymatic activity in postmortem hepatic tissue

In vitro enzyme activity rates of CYP1A were estimated in PLM based on metabolite formation of paracetamol from phenacetin, and in vitro enzyme activity rates of CYP3A were estimated in PLM by metabolite formation of alpha‐hydroxymidazolam from midazolam. The enzyme activity rates were linear between 0.1 and 1.5 mg/ml PLM for both substrates, and initial reaction rates were observed for reaction times up to 20 min (data not shown). There is a complete chromatographic separation between paracetamol and the internal standard acetaminophenol, assuring no interference of these isobaric compounds.

**FIGURE 3:**
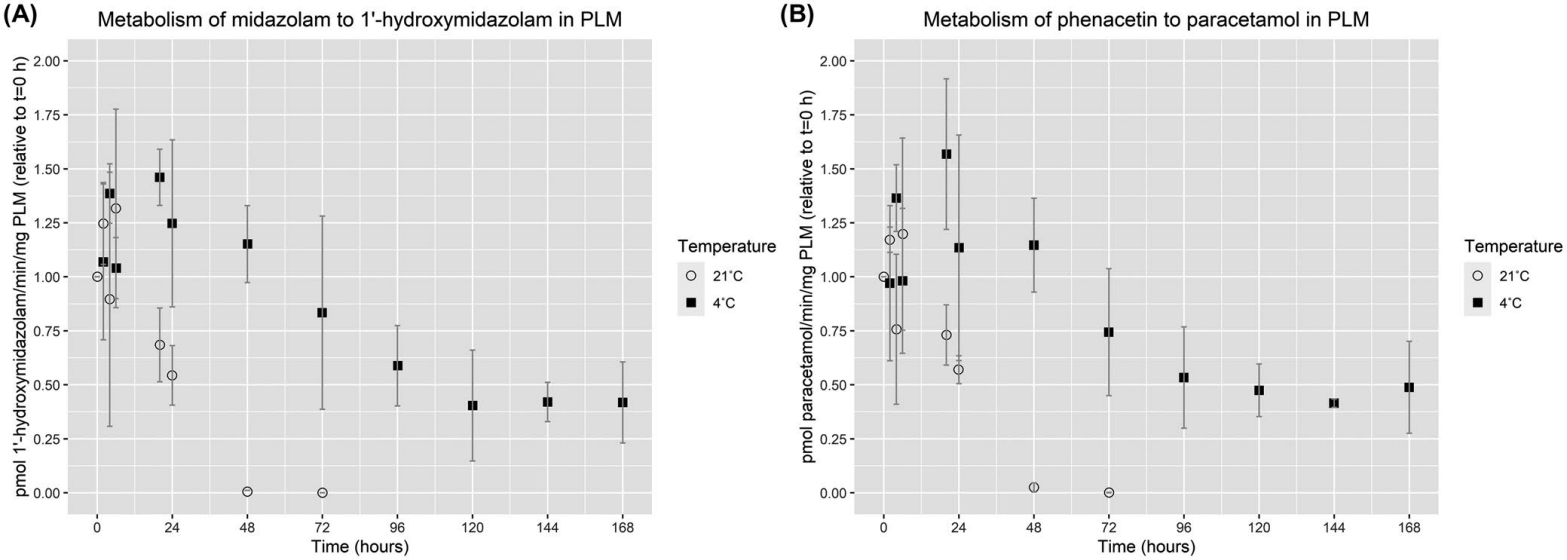
In vitro enzyme activity in postmortem porcine hepatic tissue. The mean and SD for in vitro enzyme activity rates (metabolite formation) were estimated for three livers relative to *t* = 0 h. (A) Alpha‐hydroxymidazolam formation (CYP3A). (B) Paracetamol formation (CYP1A)

The in vitro enzymatic activity rates at *t* = 0 were measured at 1600–6000 pmol/min/mg PLM for the formation of paracetamol and 1000–3800 pmol/min/mg PLM for the formation of alpha‐hydroxymidazolam for the three porcine livers (Table 1). A high degree of variability was seen in the data; therefore, the standard deviation was calculated for each time point for the three livers (Figure 3). The metabolic conversion of both metabolites declined gradually to approximately 50% in tissue incubated at 4°C for 168 h (Figure 3). When the tissue was incubated at 21°C, a 50% decrease in enzymatic activities was observed at 24 h, and at 48–72 h, the enzyme activities were completely lost (Figure 3).

## 4 DISCUSSION

To date, the stability of CYP proteins for the first 24 h postmortem remains unknown. The present study revealed that hepatic CYP1A2, CYP2D25, CYP2E1, and CYP3A29 levels are stable for 72 h or more postmortem at 4°C (Figure 2A). The stability at 4°C of MPPGL (Figure 1) and individual CYP‐isoforms (Figure 2A) followed a liver‐specific pattern, with full stability for 168 h for liver 1, and a decrease of 30%–50% for liver 2 and 3 after 72 h. At 21°C CYP1A2, CYP2D25, CYP2E1 were stable for 24 h (Figure 2B), whereas CYP3A29 had a different degradation profile with a small loss at 6 h (20%), and an approximate 50% loss at 20–24 h. At 21°C MPPGL followed the same pattern for all three livers (Figure 1). Therefore the rate at which CYP‐proteins degrade postmortem seems to vary not only between livers but also between CYP‐isoforms. The observation that CYP isoforms have individual degradation profiles at 21°C need further investigation due to the limited data presented here, but this study firmly demonstrates that temperature is an important factor for the degradation rate. Some studies have found proteins to degrade much faster than the CYP proteins investigated in this study whereas others have found certain muscle proteins to be stable for up to 10 days postmortem.^7^, ^8^, ^9^, ^10^

In nondegraded tissue, we found an average of 23.3 pmol/mg PLM CYP1A2 and 33.2 pmol/mg PLM CYP3A29 among the livers, suggesting that these CYP isoforms are relatively low abundant. CYP2D25 and CYP2E1, on the other hand, seem to be expressed at the higher levels of 61.7 pmol/mg PLM and 138.3 pmol/mg PLM, respectively (Table 1). To our knowledge, these porcine CYP proteins have only been quantified by untargeted proteomics until now.^24^, ^27^ In agreement with the present study, both papers reported CYP1A2 and CYP3A29 to be of low abundance and CYP2E1 to be highly abundant. CYP2D25 was only quantified by Achour et al. 2011, who reported approximately threefold more CYP2D25 compared to CYP2E1,^24^ whereas our study suggests that CYP2E1 is expressed in twice the abundance as CYP2D25 in porcine livers. Estimation of CYP levels in hepatic tissue has traditionally been done using an activity‐based approach. An important question is therefore also for how long the activity can be measured postmortem and how this compares to a mass spectrometry‐ based proteomics approach. To investigate this, we estimated in vitro enzymatic activity rates associated with phenacetin (CYP1A) and midazolam (CYP3A) metabolism in the same postmortem samples that were used to measure CYP protein levels. The measured CYP activity rates in nondegraded PLM (Table 1) were in agreement with the biotransformation rates of phenacetin and midazolam in PLM reported by other studies.^15^, ^28^ Furthermore, there is a correlation between the measured CYP1A2 protein levels and the in vitro enzyme activity measurements in the individual pigs, with the liver ranking liver 2 > liver 1 > liver 3 in terms of both measured CYP1A2 protein levels and CYP1A enzymatic activity measurements associated with paracetamol formation (Table 1). Liver 2 also exhibited the highest level of both CYP3A29 protein levels and alpha‐hydroxymidazolam formation (CYP3A), but for the remaining two livers, the correlation was not as clear. Half of the measured enzyme activity was lost within 24 h at 21°C for both investigated metabolic pathways. This loss in enzyme activity corresponds to the loss seen in measured CYP3A29 levels by LC–MS/MS, whereas CYP1A2 only had a slight loss at 24 h when measured by LC–MS/MS. At 48 h there was <2% enzymatic activity left, but there was still 60% CYP1A2 and 30% CYP3A29 measurable by LC–MS/MS. At 4°C the LC–MS/MS estimated CYP levels were constant for approximately 72 h, compared to a decrease of 40%–50% in enzyme activity at 96 h. Both at 4 and 21°C, it can be concluded that an LC–MS/MS‐based quantification is more robust to postmortem changes than an activity‐based approach. This is most likely because the CYP protein quantification occurs at the peptide level, whereas the enzyme activity assay requires a fully functional enzyme.

A previous study by Hansen et al. 2019 found little or no CYP1A2 and CYP3A4 activity in human postmortem HLM preparations from hepatic tissue with an estimated PMI of 24–36 h.^11^ According to the present study, activity is lost between 24 and 48 h at 21°C, which supports the findings by Hansen et al. 2019 considering the difference in study conditions.

The CYP protein quantification and the enzymatic activity measurements are both based on the yield of the microsomal protein MPPGL. In this study, we found that the MPPGL levels were between 10.9 and 14.6 mg/g liver (Table 1). The previously mentioned porcine proteomics papers reported MPPGL values of 32.6 and 36.6 mg/g liver in two adult pigs.^24^ For young pigs with an age corresponding to this study MPPGL of 11.7–15.6 mg/g liver is reported,^27^ this is in good agreement with our findings. Both studies measured cytochrome c reduction in homogenates and PLM to account for the loss of microsomal protein during PLM preparation. Only Achour et al. reported the uncorrected values of 18.1 and 30.2 mg/g liver.^24^ In our study, we did not determine the loss of microsomal protein during PLM isolation, yet our MPPGL levels are comparable to the other studies, and the variation in our PLM preparation was relatively small (CV = 10.8%–13.4%).

A limitation of this study is the in vitro postmortem degradation as opposed to whole‐body postmortem degradation. However, since the experimental setup required continuous sample collection, the skin barrier would have been breached, and air and surface bacteria would have been introduced into the wound. It is therefore questionable that the use of a whole pig body would have greatly improved the relevance of this study, and we, therefore, found the in vitro degradation setup sufficient for the purpose of this study.

The timespan from death to when an autopsy is performed varies greatly between countries and between different specific cases. Not only the temperature at the place of death and PMI, but also storage conditions until autopsy will be important for the decay of postmortem tissue. Hepatic CYP quantification will therefore only make sense in selected cases.

Hansen et al. 2019 showed that quantification of human CYP isoforms is possible in postmortem tissue, but the fundamental question regarding stability in the time period from death to 24–36 h was unanswered.^11^ The current study investigated stability from *t* = 0 h and revealed that three out of four examined CYP isoforms are stable for at least 24 h at 21°C. This finding is supported by Hansen et al. 2019 who in 50 postmortem samples found levels of human CYP1A2 and CYP3A4 comparable to reference values. These findings point to a role of postmortem tissue as a source of proteomic data. But even though the four investigated porcine CYP isoforms exhibited high sequence identity with their corresponding human CYP‐isoforms they are still different, and it is apparent that the results can not be used as a definite indicator for the postmortem stability of the human CYP isoforms. Instability of the CYPs is expected over time which will cause decreased levels in postmortem tissue. Therefore further work validating the use of human postmortem tissue is needed. We suggest that genotyping in combination with CYP‐quantification could provide insight into the validity of quantification results of postmortem human hepatic tissue, e.g., for CYP2D6 the amount of CYP‐protein is related to the number of functional alleles.^29^ This could further be supplemented by the metabolic ratio between parent compounds and metabolites for phenotypic pathways in relation to measured CYP‐levels. Known differences in CYP‐levels across subpopulations could also be used to validate the approach e.g., CYP2E1 is induced in alcoholics^30^ and examining the CYP2E1 levels in alcoholics versus nonalcoholics is one out of many possibilities to validate the usability of postmortem tissue. Also, the development of methods to estimate the degradation degree of a given hepatic sample would be important to assure acceptable results from postmortem tissue. Even if only selected postmortem cases can be used for CYP quantification, the number of autopsies performed each year will make it possible to accumulate sufficient cases over time. The virtual subpopulation or individual can further be informed by physiological data noted at autopsy e.g., height, body weight, the weight of the liver—heart—brain—lungs—kidneys, and diseases and medication history.

In this study, we only investigated the stability of CYP enzymes but it is obvious that information regarding the stability of other proteins involved in drug elimination like UGTs, and transporters would be interesting as well. Such a broad investigation was out of the scope of this work.

In conclusion, we found that postmortem CYP levels quantified by LC–MS/MS for 3 out of 4 CYP isoforms were comparable to antemortem CYP levels for 24 h if the tissues have been stored at 21°C and for 3 days if the tissue has been stored at 4°C for all four CYP isoforms. The use of readily available postmortem specimens for collecting information about protein abundances could increase the availability of data on CYP levels which is important to understanding drug disposition in subpopulations.

## DISCLOSURE

The authors declare no conflict of interest.

## AUTHORS CONTRIBUTIONS

Participated in research design: Pedersen, Hansen, Hasselstrøm, and Jornil. Conducted experiments: *Pedersen*. Contributed new reagents or analytic tools: Pedersen, Hansen, Hasselstrøm, and Jornil. Performed data analysis: Pedersen, Hansen, Hasselstrøm, and Jornil. Wrote or contributed to the writing of the manuscript: Pedersen, Hansen, Hasselstrøm, and Jornil.

## Supporting information

Table S1
Table S2

## Data Availability

The data that support the findings of this study are available from the corresponding author upon reasonable request.

## References

[1] Johnson TN , Rostami‐Hodjegan A , Tucker GT . Prediction of the clearance of eleven drugs and associated variability in neonates, infants and children. Clin Pharmacokinet. 2006;45(9):931‐956. 16928154 10.2165/00003088-200645090-00005

[2] Harnisch L , Shepard T , Pons G , Della PO . Modeling and simulation as a tool to bridge efficacy and safety data in special populations. CPT: Pharmacomet Syst Pharmacol. 2013;2(2):28. 10.1038/psp.2013.6 PMC3600759 23835939

[3] Zhuang X , Lu C . PBPK modeling and simulation in drug research and development. Acta Pharmaceutica Sinica B. 2016;6(5):430‐440. 27909650 10.1016/j.apsb.2016.04.004 PMC5125732

[4] Krogstad V , Peric A , Robertsen I , et al. Correlation of body weight and composition with hepatic activities of cytochrome P450 enzymes. J Pharm Sci. 2021;110(1):432‐437. 33091408 10.1016/j.xphs.2020.10.027

[5] Isoherranen N , Thummel KE . Drug metabolism and transport during pregnancy: how does drug disposition change during pregnancy and what are the mechanisms that cause such changes? Drug Metab Dispos. 2013;41(2):256‐262. 23328895 10.1124/dmd.112.050245 PMC3558867

[6] Verbeeck RK . Pharmacokinetics and dosage adjustment in patients with hepatic dysfunction. Eur J Clin Pharmacol. 2008;64(12):1147. 18762933 10.1007/s00228-008-0553-z

[7] ElHajj Z , Cachot A , Müller T , Riederer IM , Riederer BM . Effects of postmortem delays on protein composition and oxidation. Brain Res Bull. 2016;121:98‐104. 26791740 10.1016/j.brainresbull.2016.01.005

[8] Choi K‐M , Zissler A , Kim E , et al. Postmortem proteomics to discover biomarkers for forensic PMI estimation. Int J Legal Med. 2019;133(3):899‐908. 30864069 10.1007/s00414-019-02011-6 PMC6469664

[9] Ehrenfellner B , Zissler A , Steinbacher P , Monticelli FC , Pittner S . Are animal models predictive for human postmortem muscle protein degradation? Int J Legal Med. 2017;131(6):1615‐1621. 28721468 10.1007/s00414-017-1643-1 PMC5635072

[10] Pittner S , Monticelli FC , Pfisterer A , et al. Postmortem degradation of skeletal muscle proteins: a novel approach to determine the time since death. Int J Legal Med. 2016;130(2):421‐431. 26041514 10.1007/s00414-015-1210-6

[11] Hansen J , Palmfeldt J , Pedersen KW , et al. Postmortem protein stability investigations of the human hepatic drug‐metabolizing cytochrome P450 enzymes CYP1A2 and CYP3A4 using mass spectrometry. J Proteomics. 2019;194:125‐131. 30529742 10.1016/j.jprot.2018.11.024

[12] Schelstraete W , Devreese M , Croubels S . Storage stability study of porcine hepatic and intestinal cytochrome P450 isoenzymes by use of a newly developed and fully validated highly sensitive HPLC‐MS/MS method. Anal Bioanal Chem. 2018;410(6):1833‐1843. 29327113 10.1007/s00216-017-0839-z

[13] Soucek P , Zuber R , Anzenbacherova E , Anzenbacher P , Guengerich FP . Minipig cytochrome P450 3A, 2A and 2C enzymes have similar properties to human analogs. BMC Pharmacol. 2001;1:11. 11737866 10.1186/1471-2210-1-11 PMC60991

[14] Skaanild MT . Porcine cytochrome P450 and metabolism. Curr Pharm Des. 2006;12(11):1421‐1427. 16611125 10.2174/138161206776361183

[15] Schelstraete W , Clerck LD , Govaert E , et al. Characterization of porcine hepatic and intestinal drug metabolizing CYP450: comparison with human orthologues from a quantitative, activity and selectivity perspective. Sci Rep. 2019;9(1):9233. 31239454 10.1038/s41598-019-45212-0 PMC6592956

[16] Zamaratskaia G , Zlabek V . EROD and MROD as markers of cytochrome P450 1A activities in hepatic microsomes from entire and castrated male pigs. Sensors. 2009;9(3):2134‐2147. doi:10.3390/s90302134 22574004 PMC3345832

[17] Rasmussen MK , Zamaratskaia G , Ekstrand B . Comparable constitutive expression and activity of cytochrome P450 between the lobes of the porcine liver. Toxicol Vitro. 2014;28(7):1190‐1195. doi:10.1016/j.tiv.2014.06.002 24952075

[18] Anzenbacher P , Soucek P , Anzenbacherova E , et al. Presence and activity of cytochrome P450 isoforms in minipig liver microsomes. Comparison with human liver samples. Drug Metab Dispos. 1998;26(1):56‐59. 9443853

[19] Rasmussen MK , Zamaratskaia G , Ekstrand B . Gender‐related differences in cytochrome P450 in porcine liver–implication for activity, expression and inhibition by testicular steroids. Reprod Domest Anim. 2011;46(4):616‐623. doi:10.1111/j.1439-0531.2010.1714.x 21091800

[20] Gerber SA , Rush J , Stemman O , Kirschner MW , Gygi SP . Absolute quantification of proteins and phosphoproteins from cell lysates by tandem MS. Proc Natl Acad Sci USA. 2003;100(12):6940‐6945. doi:10.1073/pnas.0832254100 12771378 PMC165809

[21] Barter ZE , Bayliss MK , Beaune PH , et al. Scaling factors for the extrapolation of in vivo metabolic drug clearance from in vitro data: reaching a consensus on values of human microsomal protein and hepatocellularity per gram of liver. Curr Drug Metab. 2007;8(1):33‐45. 17266522 10.2174/138920007779315053

[22] Hesselager MO , Codrea MC , Sun Z , et al. The Pig PeptideAtlas: a resource for systems biology in animal production and biomedicine. Proteomics. 2016;16(4):634‐644. doi:10.1002/pmic.201500195 26699206 PMC4786621

[23] MacLean B , Tomazela DM , Shulman N , et al. Skyline: an open source document editor for creating and analyzing targeted proteomics experiments. Bioinformatics. 2010;26(7):966‐968. doi:10.1093/bioinformatics/btq054 20147306 PMC2844992

[24] Achour B , Barber J , Rostami‐Hodjegan A . Cytochrome P450 Pig liver pie: determination of individual cytochrome P450 isoform contents in microsomes from two pig livers using liquid chromatography in conjunction with mass spectrometry. Drug Metab Dispos. 2011;39(11):2130‐2134. doi:10.1124/dmd.111.040618 21795467

[25] Harding SD , Sharman JL , Faccenda E , et al. The IUPHAR/BPS Guide to PHARMACOLOGY in 2018: updates and expansion to encompass the new guide to IMMUNOPHARMACOLOGY. Nucleic Acids Res. 2018;46(D1):D1091‐D1106. doi:10.1093/nar/gkx1121 29149325 PMC5753190

[26] Alexander SPH , Fabbro D , Kelly E , et al. THE CONCISE GUIDE TO PHARMACOLOGY 2019/20: enzymes. Cytochrome P450. Br J Pharmacol. 2019;176(S1):S297‐S396. 31710714 10.1111/bph.14752 PMC6844577

[27] Millecam J , De Clerck L , Govaert E , et al. The ontogeny of cytochrome P450 enzyme activity and protein abundance in conventional pigs in support of preclinical pediatric drug research. Front Pharmacol. 2018;9:470. doi:10.3389/fphar.2018.00470 29867477 PMC5960725

[28] Hosagrahara VP , Hansen LK , Remmel RP . Induction of the metabolism of midazolam by rifampin in cultured porcine hepatocytes: preliminary evidence for CYP3A isoforms in pigs. Drug Metab Dispos. 1999;27(12):1512‐1518. 10570035

[29] Langenfeld E , Zanger UM , Jung K , Meyer HE , Marcus K . Mass spectrometry‐based absolute quantification of microsomal cytochrome P450 2D6 in human liver. Proteomics. 2009;9(9):2313‐2323. doi:10.1002/pmic.200800680 19402041

[30] Lu Y , Cederbaum AI . CYP2E1 and oxidative liver injury by alcohol. Free Radic Biol Med. 2008;44(5):723‐738. doi:10.1016/j.freeradbiomed.2007.11.004 18078827 PMC2268632

