# Supplementary material for: Stability investigations of cytochrome P450 (CYP) enzymes immediately after death in a pig model support the applicability of postmortem hepatic CYP quantification
Source: Pharmacol Res Perspect. 2021 Sep 3;9(5):e00860. doi: 10.1002/prp2.860 (PMC8415216; doi:10.1002/prp2.860)
Supplement: Supplementary file 1 — Table S1 Table S2 [file PRP2-9-e00860-s001.docx]

**Supplementary Data**

**Stability investigations of cytochrome P450 (CYP) enzymes immediately after death in a pig model support the applicability of postmortem hepatic CYP quantification**

Kata W. Pedersen, Jakob Hansen, Jørgen B. Hasselstrøm and Jakob R. Jornil

*Department of Forensic Medicine, Aarhus University, Aarhus, Denmark.*

**Table S1.** **Target porcine CYP proteins and unique tryptic peptides**. Overview of the porcine CYP proteins investigated in this study and the unique tryptic peptides used for the mass spectrometry-based quantification.

| Porcine  CYP | Accession number^1^ | Sequence Identity^2^ | Human equivalent | Porcine target  peptide sequence | Position  (start-end) |
| --- | --- | --- | --- | --- | --- |
| **CYP1A2** | F1SJ26 | 82% | CYP1A2 | YLPSPTLQR | 243-251 |
|  |  |  |  | ELDTVIGR | 346-353 |
| **CYP2D25** | O46658 | 78% | CYP2D6 | DLAQPPR | 265-271 |
|  |  |  |  | GTTLITNLTSVLK | 394-406 |
| **CYP2E1** | P79383 | 79% | CYP2E1 | FIDLIPSNLPHEATR | 359-373 |
|  |  |  |  | GVIFNNGPTWR | 113-123 |
| **CYP3A29** | P79401 | 77% | CYP3A4/5 | SSVNFFTK | 243-250 |
|  |  |  |  | DTINPYTYLPFGTGPR | 425-440 |

^1^ Entry to [www.uniprot.org](http://www.uniprot.org).

^2^ The sequence identities are based on EMBOSS pairwise sequence alignment.

**Table S2: Transitions for LC-MS/MS quantification.** The tryptic peptides were manually tuned by direct infusion, and evaluated in regards to signal intensity and linearity. The peptide with the highest intensity was used for quantification (peptide_1) and the second peptide served as a qualifier (peptide_2). Well-performing transitions were selected for quantification and qualification purposes.

| Porcine CYP | Peptide Name | Sequence | Precursor ion (m/z) | CE  (quantifier/qualifier) | Product ion (m/z) (quantifier/qualifier) |
| --- | --- | --- | --- | --- | --- |
| CYP1A2 | 1A2_1 | YLPSPTLQR | 537.8++ | 17/21 | 798.4+/614.4+ |
|  | 1A2_1_IS | YLPSPTLQ**R*** | 542.8++ | 21/17 | 808.5+/624.4+ |
|  | 1A2_2 | ELDTVIGR | 451.8++ | 12/12 | 345.2+/444.3+ |
|  | 1A2_2_IS | ELDTVIG**R*** | 456.8++ | 12/12 | 355.2+/555.4+ |
|  |  |  |  |  |  |
| CYP2D25 | 2D25_1 | DLAQPPR | 398.7++ | 12/12 | 369.2+/568.3+ |
|  | 2D25_1_IS | DLAQPP**R*** | 403.7++ | 12/12 | 379.2+/578.3+ |
|  | 2D25_2 | GTTLITNLTSVLK | 680.9++ | 22/20 | 875.5+/373.2+ |
|  | 2D25_2_IS | GTTLITNLTSVL**K*** | 684.9++ | 22/22 | 883.5+/373.2+ |
|  |  |  |  |  |  |
| CYP2E1 | 2E1_1 | FIDLIPSNLPHEATR | 862.0++ | 31/39 | 489.3+/376.2+ |
|  | 2E1_1_IS | FIDLIPSNLPHEAT**R*** | 867.0++ | 29/29 | 489.3+/376.2+ |
|  | 2E1_2 | GVIFNNGPTWR | 630.8++ | 19/19 | 991.5+/844.4+ |
|  | 2E1_2_IS | GVIFNNGPTW**R*** | 635.8++ | 19/19 | 1001.5+/854.4+ |
| CYP3A29 | 3A29_1 | SSVNFFTK | 465.2++ | 14/16 | 755.4+/656.3+ |
|  | 3A29_1_IS | SSVNFFT**K*** | 469.2++ | 16/16 | 763.4+/664.4+ |
|  | 3A29_2 | DTINPYTYLPFGTGPR | 906.5++ | 30/30 | 731.4+/844.5+ |
|  | 3A29_2_IS | DTINPYTYLPFGTGP**R*** | 911.5++ | 30/30 | 741.4+/854.5+ |

**R***: isotopically labeled arginine (^13^C_6_^15^N_4_, +10 Da)

**K***: isotopically labeled lysine (^13^C_6_^15^N_2_, +8 Da)
